# New York College of Veterinary Surgeons

**Published:** 1890-09

**Authors:** 


*New York College of Veterinary Surgeons.*—The New York College of Veterinary Surgeons has constructed a new pathological museum and has enlarged its dissecting room. Professor W. R. Ballou has published a Quiz Compend of Veterinary Anatomy.

